# Synthetic cannabinoids awareness among patients with opioid use disorder in Serbia – A survey based cross-sectional pilot study

**DOI:** 10.3389/fpsyt.2023.987726

**Published:** 2023-03-07

**Authors:** Vesna Mijatović Jovin, Isidora Dickov, Dragana Ratković, Aleksandra Dickov, Ana Tomas

**Affiliations:** ^1^Department of Pharmacology, Toxicology and Clinical Pharmacology, Faculty of Medicine, University of Novi Sad, Novi Sad, Serbia; ^2^Department of Gynecology and Obstetrics, Faculty of Medicine, University of Novi Sad, Novi Sad, Serbia; ^3^Clinic for Gynecology and Obstetrics, Clinical Center of Vojvodina, Novi Sad, Serbia; ^4^Department of Psychiatry and Psychological Medicine, Faculty of Medicine, University of Novi Sad, Novi Sad, Serbia; ^5^Clinic for Psychiatry, Clinical Center of Vojvodina, Novi Sad, Serbia

**Keywords:** synthetic cannabinoids, opioid use disorder, use, awareness, predictive factors

## Abstract

**Introduction:**

There is limited data on the awareness and use of synthetic cannabinoids (SCs) in high-risk population in Serbia, despite SCs becoming more and more common at illicit drug market.

**Aim:**

This pilot study aimed to examine the awareness and prevalence of use of SCs in patients with an opioid-use disorder and to identify patient characteristics and other factors associated with SCs use.

**Patients and methods:**

This cross-sectional study was conducted at the Clinic for Psychiatry, Clinical Center Vojvodina, Serbia, the largest tertiary health care institution in this region of the country. All patients hospitalized due to the treatment of opioid dependence during November and December 2017 were included (response rate 100%), and filled-out an anonymous questionnaire specifically developed for the purpose of this study. Differences between patients reporting SCs use and those who did not were compared using chi-square test with values of *p* < 0.05 were considered significant.

**Results:**

Out of 64 patients (median age 36.37 years), one third (32.81%) reported using SCs. Socio-demographic characteristics of the subjects were not associated with SCs use. There were differences in the most common sources of information reported between the SCs users and non-users. Majority of SCs users (76.0%) were informed about SCs through friends, compared with just 26.0% of non-users (<0.001). Nearly all study participants (93.8%) were daily tobacco users. The share of respondents reporting alcohol and marihuana use was significantly higher among the SCs users (52.0% vs. 20.9%, *p* = 0.011 and 15.6% vs. 12.5%, *p* = 0.015), respectively. Higher share of SCs users used multiple psychoactive substances (38.1% vs. 16.3%), and this difference was statistically significant (*p* = 0.047). The most commonly reported adverse effect of SCs among users included dry mouth (81.0%), trouble thinking clearly (52.4%) and panic attacks (52.4%).

**Conclusion:**

Understanding the awareness and use of SCs among high-risk drug users, as well as associated factors can help improve substance-use disorder treatment in our setting. Educational activities targeting public are urgently needed to raise awareness on SCs, considering that social contacts are the main sources of information on SC for this vulnerable population. Users of SCs have also reported using other psychoactive substances more often, and this calls for a holistic approach addressing multiple factors to improve substance-use treatment in our setting.

## Introduction

The United Nations Office for Drugs and Crime has defined NPS (new psychoactive substances) as “substances of abuse, either in a pure form or a preparation, that are not controlled by the 1961 Single Convention on Narcotic Drugs or the 1971 Convention on Psychotropic Substances, but which may pose a public health threat” (1, 2). According to the distinguished experts in the field, NPS are also defined as narcotic drugs or psychotropic substances made available or used from the early to mid-2000s for their psychoactive properties (3).

The appearance of NPS has raised considerable concern at international level. One of the reasons could be a high number of substances identified by the Early Warning System every year - at the end of 2021 the EMCDDA (European Monitoring Centre for Drugs and Drug Addiction) was monitoring around 880 NPS, 52 of which were first reported in Europe in 2021 (2). In addition, a growing number of NPS consumers have been assessed by health care professionals (HCPs) with very severe clinical manifestations and unpredictable NPS associated untoward effects (4, 5). Considering their psychopathological consequences, which are of main interest for mental HCPs, NPS include: synthetic cannabinoids/cannabimimetics (SCs), new synthetic opioids, ketamine-like dissociatives, novel stimulants and novel psychedelics, prescription and over-the-counter medicines with a misusing potential (6).

The prevalence of NPS use is very difficult to estimate, especially for the countries not included in the standard world warning and monitoring systems. Scarce data available originates from analysis of calls to national poison control centers, emergency department admissions and drug use surveys (7). According to the ESPAD (European School Survey Project on Alcohol and Other Drugs) study from 2019 which collected information on NPS in general, 3.4% of the ESPAD students surveyed had tried NPS during their lifetime and 2.5% had used them in the past 12 months (8). While surveys about NPS consumption have been most commonly focused on one group of potential/real users, the NPS-transnational (NPS-t) project included 6 European countries (Germany, Hungary, Ireland, the Netherlands, Poland and Portugal) and 3 groups of NPS consumers (socially marginalized, users in nightlife settings and in online communities) (9). In the total sample, last year prevalence was by far highest for stimulant NPS, followed by psychedelic NPS and herbal blends and/or SCs, while it was lowest for dissociative NPS (9). Moreover, prevalence rate also varied across countries included in the study (9). Additionally, to investigate the use of selected NPS in general population, wastewater-based epidemiology has been applied in 14 European countries (including Serbia) during 2016 and 2017 (10). Methcathinone was most frequently detected (>65% of the cities), followed by mephedrone (>25% of the cities), and only mephedrone, methcathinone and methylone were found in both years (10). Also, this study confirms that NPS use in Europe is much lower than the use of classical psychoactive drugs (10). On the other hand, lived experience of people who use NPS as well as people who provide harm reduction services were main sources of information for the study conducted by Kurcevič et al. in 6 Euroasian countries, including Serbia (11).

The Early Warning System in Serbia has been active since 2016. An increase in the prevalence of synthetic stimulants and other NPS use has been reported within EMCDDA publication next year (2017) (12). The study pointed out first clinical and analytical experience of the Serbian National Poison Control Centre with SCs, as representatives of NPS, was published in 2018 (13). Even though the inhalation of SCs is intended to mimic the psychotropic effects of cannabis (mostly euphoric effects and relaxation), SCs undesirable effects are unpredictable and more severe (e.g., cardiac arrest, liver toxicity, kidney failure, seizures) than those provoked by phytocannabinoids (14). The most prominent psychiatric and neurological adverse effects are: auditory/visual hallucinations, anxiety and agitation, paranoia, mood swings, suicidal ideation and attempts, thought disorganization and delirium (4, 6, 15). Even a peculiar “synthetic” psychosis, designated “Spiceophrenia,” has been described as a chronic psychopathological symptom of SCs consumption (5). Finally, SCs use could lead to dependence, tolerance and withdrawal phenomena (16).

Evaluating awareness and attitudes of future HCPs in Serbia regarding NPS, Mijatović Jovin et al. in the most recent paper emphasized the necessity for improving theoretical and clinical lectures about NPS for prospective doctors in the context of their future role in the prevention and treatment of NPS overdose and addiction (17).

To the best of our knowledge, there is no available data on the awareness and use of SCs in patients admitted to drug detoxification treatment in Serbia. This survey based cross-sectional pilot study aimed to examine the awareness and use of SCs in patients with an opioid-use disorder and to identify patient characteristics and other factors associated with SCs use.

## Method

This cross-sectional, observational study was conducted at the Department for substance-related and addictive disorders of the Clinic for Psychiatry Clinical Center Vojvodina. All patients hospitalized for the treatment of opioid dependence (F11.2 according to International Classification of Diseases, 10th edition – ICD-10) during November and December 2017 were asked to participate, and all have accepted (response rate 100.0%).

The participants were informed that survey completion was voluntary, confidential, that no personal information would be collected/processed and published, and that no compensation would be provided for their potential participation. Study personnel assessed participants’ understanding about the study by asking whether they understood the nature and purpose of the study, the informed consent procedure, and if there were any questions prior to survey administration. After obtaining consent for participation, the survey was started.

The investigation was approved by the Ethics Committee of the Clinical Center of Vojvodina as well as the Ethics Committee of the Faculty of Medicine, University of Novi Sad.

Based on the literature, recommended minimum sample size for pilot and feasibility studies is between 24 and 50 participants (18–20). Taking this into account, with a sample size of 50, we would have been able to estimate a prevalence of SC use of 30% within a 95% confidence interval of ±13%.

Items of the study included questions about demographics and past and current (past 30-day) substance consumption (tobacco, alcohol, marijuana, and other drugs). The survey also inquired about (1) source of information regarding SCs, (2) SCs product use and its frequency (pattern of use), (3) undesirable subjective effects of SCs products (risk and consequences of SCs use), and (4) types of SCs used and common slang names. Questionnaire used is available in the Supplementary material.

Descriptive statistics, Chi-square test for categorical variables and t-test for continuous variables were used for the analysis. Analyses were performed IBM SPSS Statistics V.22 software. A *p* level < 0.05 was considered statistically significant.

## Results

Out of 64 patients included in the study, one-third (21, 32.81%) reported ever using SCs. One third of the SCs (28.57%) consumers used SCs daily. Majority of the respondents (53, 82.81%) identified as male. The median age of respondents was 36.37 years, with no significant difference in age between SCs users and non-users (*F* = 0.007, *p* = 0.93). Almost all (87.5%) reported completing secondary school. Most (64.06%) were currently employed, and the rest reported being unemployed and looking for job. Most of the survey subjects (81.25%) were from urban areas. Regarding relationship status, one half of the participants were single (51.56%). There were no differences in demographic characteristics between those reporting having ever used SCs and non-users (Table 1).

**Table 1:** Socio-demographic characteristics of the respondents (*N* = 64).

Parameter	No	%	*χ*^2^*	Value of *p*
*Gender*	64	100	0.963	0.326
male	53	82.81		
female	11	17.19		
*Education*	64	100	7.064	0.216
less than high school	7	10.94		
high school or equivalent	56	87.5		
college and above	1	1.56		
*Living arrangements*	64	100	3.032	0.553
married/cohabiting/civil union	25	39.06		
single	33	51.56		
divorced/widowed/separated	6	9.38		
*Employment status*	64	100	5.341	0.148
employed full-time/part time	21	32.81		
temporary jobs	20	31.25		
unemployed	23	35.94		
*Residential area*	64	100	1.746	0.186
urban	52	81.25		
rural	12	18.75		
*Family type*	64	100	4.307	0.366
nuclear	23	35.94		
joint	32	50.00		
alone	9	14.06		
*Living*				
with young children	22	34.38	3.032	0.553
with opiate addict	9	14.06	0.533	0.465

*users vs. non-users.

Over one-third of the participants (42.0%) were informed about SCs by a friend, while 37.0% of respondents were exposed to information regarding SCs by traditional and social media (Table 2). There were differences in the most common sources of information reported between the SCs users and non-users. Majority of SCs users (76.0%) were informed about SCs through friends, compared with just 26.0% of non-users. Higher share of non-users reported social media and internet as sources of information in comparison with SCs users (37.0% vs. 10.0%). Also, a third of non-users reported never having heard about SCs. Respondents’ habits on past and current substance use, and differences between SCs users and non-users are reported in Table 3. Nearly all study participants (93.8%) were daily tobacco users, with a slightly higher percentage of smokers among the non-user group (83.7% vs. 81.0%, *p* = 0.783). Overall, approximately one third reported alcohol and marijuana use, 31.3 and 28.1%, respectively. However, the share of respondents reporting alcohol and marijuana use was significantly higher among the SCs users (52.4% vs. 20.9%, *p* = 0.011 and 15.6% vs. 12.5%, *p* = 0.015, respectively). Higher share of SCs users used multiple psychoactive substances (38.1% vs. 16.3%), and this difference was statistically significant as well (*p* = 0.047).

**Table 2:** Source of information regarding SCs.

Source	Users	Non-users	Total	*χ*^2^, Value of *p*
Friends	16 (76.0%)	11 (26.0%)	27 (42.0%)	19.564, *<0.001*
Social media and internet	2 (10.0%)	16 (37.0%)	18 (28.0%)	
TV, radio, newspapers	3 (14.0%)	3 (7.0%)	6 (9.0%)	
Have not heard about SC	0 (0.0%)	13 (30.0%)	13 (20.0%)	
Total	21 (100.0%)	43 (100.0%)	64 (100.0%)	

**Table 3:** Past and current substance use.

Substance	SCs users (*N*, %)	Non-users (*N*, %)	Total (*N*, %)	*χ*^2^	Value of *p*
Tobacco	17 (81.0%)	36 (83.7%)	60 (93.8%)	0.076	0.783
Alcohol	11 (52.4%)	9 (20.9%)	20 (31.3%)	6.496	0.011
Marijuana	10 (15.6%)	8 (12.5%)	18 (28.1%)	5.876	0.015
Polytoxicomania	8 (38.1%)	7 (16.3%)	15 (23.4%)	4.068	0.047
Total	21 (100.0%)	43 (100%)	64 (100.0%)		

Ninety (90.48%) out of 21 SCs users thought that “*Usage of SCs is more dangerous than consumption of marijuana*,” while slightly over half of non-users (55.81%) agreed with this claim, and this difference was statistically significant (*χ*^2^ = 7.885, *p* = 0.023).

**Figure 1:**
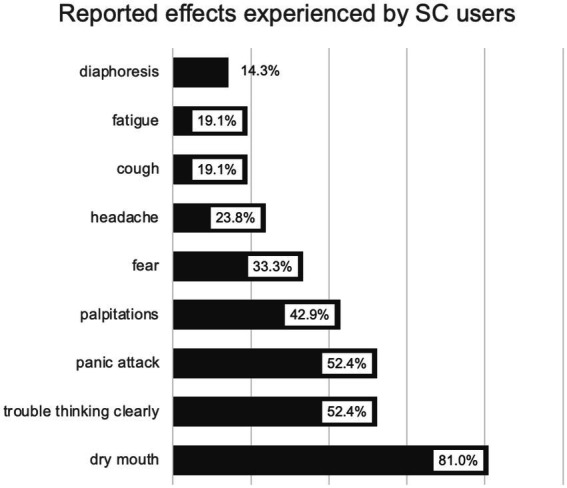
Reported adverse effects among SCs users (*N* = 21).

The most reported adverse effects associated with SCs use were dry mouth (81.0%), followed by trouble thinking clearly (52.4%) and panic attack (52.4%) (Figure 1).

Survey subjects reported 29 different names of new psychoactive drugs available on the Serbian market, but just 3 (10.34%) of them were actually SCs (mamba, k2, black sabath).

## Discussion

The present study identified prevalence of SCs use and associated factors among a vulnerable population of persons being treated for opioid-use disorder in order to bring light to this understudied topic considering their increasing availability and number of SCs on the market. Persons with opioid-use disorder commonly abuse other psychoactive substances, including cannabis, which makes them vulnerable to use SCs as well. This is influenced by a combination of social, psychological and economic factors, in addition to biological features of the opioid and cannabinoid receptor systems. SCs, diverse compounds exhibiting high affinity for the cannabinoid receptors, are known to interact with opioids in many physiological and pathological functions, including addiction (16). A neurobiological convergence of the cannabinoid and opioid systems is apparent at both receptor and behavioral levels. Both CB1 and CB2 agonists are able to induce antinociception by increasing opioid precursors’ gene expression or *via* release of endogenous opioids (21, 22). CB2 receptors activation indirectly stimulates opioid receptors located in primary afferent pathways, implying that cannabinoids can enhance opioid effects (23). Pharmacological modulation of the opioid system can modify the effects of THC (24). SCs use in this vulnerable population can contribute to the worsening of patient’s overall prognosis and prolongation of recovery (25), as polydrug use was reported to be associated with serious injury, adverse effects on health, fatal overdose, suicidal ideas and suicide attempts (4, 26, 27). In addition, SCs are one of the NPS with the highest number of reported intoxications (28, 29). Cannabis use was also found to be a predictor of the duration of untreated psychosis, and of disease outcome (30).

In the present study, more than a third of respondents (32.81%) reported having ever used SCs, similarly to the prevalence of SCs or NPS use, reported in Italy (33.5%) (25), Germany (32%) (31) and France (29%) (32). Several patient characteristics were examined with the respect to SCs use. Our study showed that the use of SCs was not reserved exclusively for adolescent population, as often reported, with the average age of the persons reporting SCs use being 36.37 years. Evaluating health and social problems associated with recent NPS use in 6 European countries, Van Hout et al. reported that average age of marginalized, night life and online users was 33.5, 25.7, and 23.6 years, respectively (33), while Soussan et al. revealed age range between 18 and 75 years (mean 27.6 years) in a self-selected sample of international NPS users (34). In contrast to other studies (35, 36), we did not find associations between socio-demographic characteristics such as income, education or social-deprivation and SCs use, but several other factors related to the use of SCs in persons with an opioid-use disorder were confirmed.

Being marijuana, alcohol and/or polydrug user were the most important risk factors for the SCs use. These results are in line with previous findings, suggesting that SCs (and other NPS) use is associated with polysubstance abuse in opioid addicts. The classic concept of defining polysubstance use as the simultaneous consumption of 3–4 “classic” drugs (e.g., opioids, amphetamines, cocaine, and cannabinoids) (37) should be reassessed to also include the use of NPS (38), in light of their growing uptake in the illicit drug market. A recent study based on the urine analysis reported the prevalence of polysubstance use of 66.3% in patients with opioid use disorder, with detection of NPS in more than 25% of samples (39). Differentiating by type of NPS, new synthetic opioids were most commonly determined (8.6%), while NPS cannabinoid type was detected in 3.2% of samples (39). Findings by Larabi et al., where 54% of NPS users had been exposed to more than one NPS in the same time period, mostly in combination with cocaine, amphetamines and opioids (32), gives further support for the problem of polydrug use associated with the NPS. In addition, SCs are not merely substitutes for older “classic” psychoactive substances, but rather represent an inseparable part of the drug repertoires. A study by Elliott et al. showed that marijuana and SCs are not commonly used interchangeably by the same individuals, implying either that the substance effects are sufficiently different, or that SCs are used out of a perceived necessity, not preference (40). In Serbia, NPS were commonly consumed in combination with amphetamines, cocaine, ecstasy, alcohol and cannabis (11). Reasons for drug combinations were: to prolong or amplify the drug’s effects, to add a new effect and to alleviate drug hangovers (11). Acute side effects reported strongly support those described in the literature and included dry mouth, trouble thinking clearly, panic attack, palpitations and fear (6, 11, 13, 14). SCs users from our study were more aware of dangers associated with the SCs consumption than non-users. On the other hand, being asked to report names of SCs available in Serbia, opioid addicts in our survey reported 29 different names, of which only 10.34% belong to SCs. In a qualitative study by Kurcevič et al. intended to identify patterns of NPS use in 6 Euroasian countries, respondents from Serbia were not entirely clear what constituted “new psychoactives” and numbered several substances, including SCs, as being “new” in the context of Serbian drug scene (11).

This pilot study has estimated prevalence of SCs use in this vulnerable population, and identified opportunities for larger scale research. With high willingness of patients to participate in study of this type, hospital setting seems a suitable one for recruitment of this population. Other that opening venue for further research, study identified several opporutnites to improve patient care, including offering a basis for preventative strategies. Firstly, the HCPs involved in the care for the patients with opioid use disorder should also be aware of the possibility of patients using SCs, as these appear to be frequent concomitants to the conventional drugs of abuse (41). This should be included when developing treatment plans tailored to specific challenges of each individual patient to support treatment and recovery. To allow for harm reduction, patients should be informed about the dangers of such practice and the fact that these combinations can produce unwanted and unpredictable effects. It was identified that most of the SCs users in the present study were acquainted with SCs through social-contacts. Patients who did not report SCs use listed internet as the main source of information on the NPS. According to Van Hout et al. younger drug users (average age of 23.6 years) are more active online, while marginalized, high-risk users of NPS obtain their drugs from friends or dealers as opposed to the Internet (33). In addition, high-risk group was older (average age 33.5 years) and among the NPSs, listed herbal blends and/or SCs as the those most commonly used (33). This calls for broader educational health communication campaigns about the risks of polydrug use and dangers of the NPS, targeting population at risk to decrease misconceptions and provide reliable information.

## Conclusion

Understanding the awareness and use of SCs among high-risk drug users, as well as associated factors, could enable more effective prevention and harm reduction within this vulnerable marginalized population. Educational activities targeting public are urgently needed to raise awareness on SCs, considering that social contacts are the main sources of information for this vulnerable population. Users of SCs have also reported using other psychoactive substances more often, and this calls for a holistic approach addressing multiple factors to improve substance-use treatment in our setting.

## Limitations

This study had several limitations that need to be mentioned. As it was based on a survey of patients currently being treated, this might have resulted in selection bias. As with any self-reported measure, recall and reporting bias cannot be excluded, which might have resulted in under-or over-reporting of SCs use. Study included a low number of participants for a disorder with a difficult to estimate prevalence. However, even despite the limitations of this survey, findings identify several problem areas in a difficult-to-sample population. A follow-up study, including the data from health-care records in addition to self-reported measures, as well as larger sample is warranted, which would make it necessary to include other treatment centers in the country.

## Data availability statement

The original contributions presented in the study are included in the article/Supplementary material, further inquiries can be directed to the corresponding author.

## Ethics statement

The studies involving human participants were reviewed and approved by Ethics Committee of the Clinical Center of Vojvodina and the Ethics Committee of the Faculty of Medicine, University of Novi Sad. Written informed consent for participation was not required for this study in accordance with the national legislation and the institutional requirements.

## Author contributions

All authors listed have made a substantial, direct, and intellectual contribution to the work and approved it for publication.

## Funding

The Ministry of Education, Science and Technological Development of the Republic of Serbia (grant numbered 451-03-68/2022-14/200114) supported this research work.

## Conflict of interest

The authors declare that the research was conducted in the absence of any commercial or financial relationships that could be construed as a potential conflict of interest.

## Publisher’s note

All claims expressed in this article are solely those of the authors and do not necessarily represent those of their affiliated organizations, or those of the publisher, the editors and the reviewers. Any product that may be evaluated in this article, or claim that may be made by its manufacturer, is not guaranteed or endorsed by the publisher.

## References

[1] United Nations Office on Drugs and Crime. Global smart update 2016. Available at: https://www.unodc.org/documents/scientific/Global-SMARTUpdate-2016-vol-16.pdf (Accessed August 23, 2022).

[2] European Monitoring Centre for Drugs and Drug Addiction. European drug report 2022: Trends and developments. Luxembourg: Publications Office of the European Union (2022).

[3] Peacock A Bruno R Gisev N Degenhardt L Hall W Sedefov R. New psychoactive substances: Challenges for drug surveillance, control, and public health responses. Lancet. (2019) 394:1668–84. doi: 10.1016/S0140-6736(19)32231-7 31668410

[4] Chiappini S Mosca A Miuli A Santovito MC Orsolini L Corkery JM. New psychoactive substances and suicidality: A systematic review of the current literature. Medicina. (2021) 57:580. doi: 10.3390/medicina57060580 34204131 PMC8226910

[5] Orsolini L Chiappini S Papanti D De Berardis D Corkery JM Schifano F. The bridge between classical and "synthetic"/chemical psychoses: Towards a clinical, psychopathological, and therapeutic perspective. Front Psych. (2019) 10:851. doi: 10.3389/fpsyt.2019.00851 PMC6896660 31849723

[6] Schifano F Napoletano F Chiappini S Guirguis A Corkery JM Bonaccorso S. New/emerging psychoactive substances and associated psychopathological consequences. Psychol Med. (2021) 51:30–42. doi: 10.1017/S0033291719001727 31327332

[7] Varì MR Mannocchi G Tittarelli R Campanozzi LL Nittari G Feola A. New psychoactive substances: evolution in the exchange of information and innovative legal responses in the European Union. Int J Environ Res Public Health. (2020) 17:8704. doi: 10.3390/ijerph17228704 33238595 PMC7709051

[8] ESPAD Report. Results from the European school survey project on alcohol and other drugs. Lisabon: EMCDDA (2019).

[9] Korf D Benschop A Werse B Kamphausen G Felvinczi K Dąbrowska K. How and where to find NPS users: A comparison of methods in a cross-National Survey among Three Groups of current users of new psychoactive substances in Europe. Int J Ment Health Addiction. (2021) 19:873–90. doi: 10.1007/s11469-019-0052-8

[10] Castiglioni S Salgueiro-González N Bijlsma L Celma A Gracia-Lor E Beldean-Galea MS. New psychoactive substances in several European populations assessed by wastewater-based epidemiology. Water Res. (2021) 195:116983. doi: 10.1016/j.watres.2021.116983 33721674

[11] Kurcevič E Lines R. New psychoactive substances in Eurasia: a qualitative study of people who use drugs and harm reduction services in six countries. Harm Reduct J. (2020) 17:94. doi: 10.1186/s12954-020-00448-2 33256747 PMC7703505

[12] European Monitoring Centre for Drugs and Drug Addiction. European Drug Report. Luxembourg: Publications Office of the European Union 2017.

[13] Vučinić S Kilibarda V Đorđević S Đorđević D Perković-Vukčević N Vuković-Ercegović G. Clinical and analytical experience of the National Poison Control Centre with synthetic cannabinoids. Arh Hig Rada Toksikol. (2018) 69:178–85. doi: 10.2478/aiht-2018-69-3096 29990297

[14] Cohen K Weinstein AM. Synthetic and non-synthetic cannabinoid drugs and their adverse effects – a review from public health. Front Public Health. (2018) 6:162. doi: 10.3389/fpubh.2018.00162 29930934 PMC5999798

[15] Schifano F Orsolini L Papanti D Corkery JM. Novel psychoactive substances of interest for psychiatry. World Psychiatry. (2015) 14:15–26. doi: 10.1002/wps.20174 25655145 PMC4329884

[16] Orsolini L Chiappini S Volpe U Berardis D Latini R Papanti GD. Use of medicinal cannabis and synthetic cannabinoids in post-traumatic stress disorder (PTSD): A systematic review. Medicina (Kaunas). (2019) 55:525. doi: 10.3390/medicina55090525 31450833 PMC6780141

[17] Mijatović Jovin V Skoko N Tomas A Živanović D Sazdanić D Gvozdenović N. New psychoactive substances: awareness and attitudes of future health care professionals in Serbia. Int J Environ Res Public Health. (2022) 19:14877. doi: 10.3390/ijerph192214877 36429596 PMC9691219

[18] Browne RH. On the use of a pilot sample for sample size determination. Stat Med. (1995) 14:1933–40. doi: 10.1002/sim.4780141709 8532986

[19] Sim J Lewis M. The size of a pilot study for a clinical trial should be calculated in relation to considerations of precision and efficiency. J Clin Epidemiol. (2012) 65:301–8. doi: 10.1016/j.jclinepi.2011.07.011 22169081

[20] Julious SA. Sample size of 12 per group rule of thumb for a pilot study. Pharm Stat. (2005) 4:287–91. doi: 10.1002/pst.185

[21] Spano MS Fadda P Fratta W Fattore L. Cannabinoid-opioid interactions in drug discrimination and self-administration: Effect of maternal, postnatal, adolescent and adult exposure to the drugs. Curr Drug Targets. (2010) 11:450–61. doi: 10.2174/138945010790980295 20017729

[22] Cichewicz DL. Synergistic interactions between cannabinoid and opioid analgesics. Life Sci. (2004) 74:1317–24. doi: 10.1016/j.lfs.2003.09.038 14706563

[23] Manzanares J Julian M Carrascosa A. Role of the cannabinoid system in pain control and therapeutic implications for the management of acute and chronic pain episodes. Curr Neuropharmacol. (2006) 4:239–57. doi: 10.2174/157015906778019527 18615144 PMC2430692

[24] Wiese B Wilson-Poe AR. Emerging evidence for Cannabis’ role in opioid use disorder. Cannabis Cannabinoid Res. (2018) 3:179–89. doi: 10.1089/can.2018.0022 30221197 PMC6135562

[25] Dal Farra D Valdesalici A Zecchinato G De Sandre A Saccon D Simonato P. Knowledge and use of novel psychoactive substances in an Italian sample with substance use disorders. Int J Environ Res Public Health. (2022) 19:915. doi: 10.3390/ijerph19020915 35055743 PMC8776073

[26] Hassan AN Le Foll B. Polydrug use disorders in individuals with opioid use disorder. Drug Alcohol Depend. (2019) 198:28–33. doi: 10.1016/j.drugalcdep.2019.01.031 30877954

[27] Alías-Ferri M Pellegrini M Marchei E Pacifici R Rotolo MC Pichini S. Synthetic cannabinoids use in a sample of opioid-use disorder patients. Front Psych. (2022) 13:956120. doi: 10.3389/fpsyt.2022.956120 PMC9381952 35990071

[28] European Monitoring Centre for Drugs and Drug Addiction. New psychoactive substances: global markets, global threats and the COVID-19 pandemic In: . An update from the EU early warning system. Luxembourg: Publications Office of the European Union (2020). 2020.

[29] United Nations Office on Drugs and Crime. Current NPS threats, vol. III. Vienna: UNODC Laboratory and Scientific Section (2020). 2020 p.

[30] Ricci V Martinotti G Ceci F Chiappini S Di Carlo F Burkauskas J. Duration of untreated disorder and cannabis use: an observational study on a cohort of young Italian patients experiencing psychotic experiences and dissociative symptoms. Int J Environ Res Public Health. (2021) 18:12632. doi: 10.3390/ijerph182312632 34886357 PMC8657003

[31] Specka M Kuhlmann T Sawazki J Bonnet U Steinert R Cybulska-Rycicki M. Prevalence of novel psychoactive substance (NPS) use in patients admitted to drug detoxification treatment. Front Psych. (2020) 11:569. doi: 10.3389/fpsyt.2020.00569 PMC7358402 32733288

[32] Larabi IA Fabresse N Etting I Nadour L Pfau G Raphalen JH. Prevalence of new psychoactive substances (NPS) and conventional drugs of abuse (DOA) in high risk populations from Paris (France) and its suburbs: a cross sectional study by hair testing (2012-2017). Drug Alcohol Depend. (2019) 204:107508. doi: 10.1016/j.drugalcdep.2019.06.011 31670189

[33] Van Hout MC Benschop A Bujalski M Dąbrowska K Demetrovics Z Felvinczi K. Health and social problems associated with recent novel psychoactive substance (NPS) use amongst marginalised, nightlife and online users in six European countries. Int J Ment Health Addict. (2018) 16:480–95. doi: 10.1007/s11469-017-9824-1 29674947 PMC5897487

[34] Soussan C Kjellgren A. The users of novel psychoactive substances: online survey about their characteristics, attitudes and motivations. Int J Drug Policy. (2016) 32:77–84. 27184218 10.1016/j.drugpo.2016.03.007

[35] Neicun J Yang JC Shih H Nadella P van Kessel R Negri A. Lifetime prevalence of novel psychoactive substances use among adults in the USA: sociodemographic, mental health and illicit drug use correlates. Evidence from a population-based survey 2007-2014. PLoS One. (2020) 15:e0241056. doi: 10.1371/journal.pone.0241056 33125395 PMC7598490

[36] Felvinczi K Benschop A Urbán R Van Hout MC Dąbrowska K Hearne E. Discriminative characteristics of marginalised novel psychoactive users: a transnational study. Int J Ment Health Addiction. (2020) 18:1128–47. doi: 10.1007/s11469-019-00128-8

[37] Petry NM Bickel WK. Polydrug abuse in heroin addicts: a behavioral economic analysis. Addiction. (1998) 93:321–35. doi: 10.1046/j.1360-0443.1998.9333212.x 10328041

[38] Gittins R Guirguis A Schifano F Maidment I. Exploration of the use of new psychoactive substances by individuals in treatment for substance misuse in the UK. Brain Sci. (2018) 8:58. doi: 10.3390/brainsci8040058 29601550 PMC5924394

[39] Alías-Ferri M Pellegrini M Marchei E Pacifici R Rotolo MC Pichini S. New psychoactive substances consumption in opioid-use disorder patients. Biology. (2022) 11:645. doi: 10.3390/biology11050645 35625373 PMC9138226

[40] Elliott L Haddock CK Campos S Benoit E. Polysubstance use patterns and novel synthetics: a cluster analysis from three U.S. cities. PLoS One. (2019) 14:e0225273. doi: 10.1371/journal.pone.0225273 31794586 PMC6890248

[41] Shapira B Berkovitz R Rosca P Neumark Y. Recent use of synthetic cannabinoids, synthetic opioids, and other psychoactive drug groups among high-risk drug users. J Psychoactive Drugs. (2020) 52:334–43. doi: 10.1080/02791072.2020.1754534 32345134

