# Supplementary material for: Synthetic cannabinoids awareness among patients with opioid use disorder in Serbia – A survey based cross-sectional pilot study
Source: Front Psychiatry. 2023 Mar 7;14:987726. doi: 10.3389/fpsyt.2023.987726 (PMC10028092; doi:10.3389/fpsyt.2023.987726)
Supplement: Supplementary file 1 [file Table_1.DOC]

**Suppl 1. Questionnaire – translated**

**Synthetic cannabinoids awareness among patients with opioid use disorder**

**Socio-demographic characteristics**

1. Gender:

1. Male
2. Female

2. Age (years): _____

3. Highest level of education attained:

1. Primary school
2. Secondary school
3. College
4. University
5. Other_____

4. Employment status:

| 1. Employed (full/part time) |
| --- |
| 1. Temporary jobs |
| 1. Unemployed 2. Other_____ |

5. Residential area?

1. Urban
2. Rural

6. Family type:

1. Nuclear
2. Joint
3. Alone

7. Living arrangements:

1. Married/cohabiting/civil union
2. Single

| 1. Divorced/widowed/separated |
| --- |

8. Do you live with another addict?

1. Yes
2. No

9. Do you have children?

1. Yes
2. No

**Risk behavior**

1.What substance or addiction are you concerned about?

1. Heroin
2. Cocaine
3. Marijuana
4. Amphetamines
5. Other_____

2. How old were you when you used the abovementioned substance for the first time?

1. <18 years
2. 18-25 years
3. 26-35 years
4. >35 years

3. What was the main reason for taking the substance for the first time?

1. To show off
2. Peer-pressure
3. Family issues
4. Issues at school/ work
5. Psychological reasons (dealing with depression, anxiety, insecurity)
6. Boredom
7. To have fun
8. Being curious
9. Poor knowledge of possible detrimental effects
10. Other_____

4. Do you drink alcohol?

1. Yes
2. No

5. If you drink alcohol, how often do you consume it?

1. Daily
2. Several times a week, but not daily
3. Several times per month

6. Are you using marihuana?

1. Yes
2. No

7. If you are using marihuana, how often do you consume it?

1. Daily
2. Several times a week, but not daily
3. Several times per month

8. Are you using multiple psychoactive substances, consuming them at the same time?

1. Yes
2. No

10. Are you using multiple psychoactive substances, which ones do you most often combine?

___________________________________

11. Are you a smoker?

1. Yes
2. No

Synthetic cannabinoids (SC) information

1. Are you familiar with synthetic cannabinoids?

(SK)?

1. Yes
2. Yes, but superficially
3. No

2. If you are familiar with the term synthetic cannabinoids, what was your source of information?

1. Internet
2. Acquaintances/friends
3. Family members
4. Other_____________

3. have you ever used synthetic cannabinoids?

1. Yes
2. No

4. If you did, how often have you used them?

1. Every day
2. Several times a week, but not every day
3. Several times a month
4. Other__________________

5. If you have used synthetic cannabinoids, have you had any of the side effects (multiple answers possible)?

1. Confusion and disorientation
2. Headache
3. Dry mouth
4. Fear
5. Rapid heartbeat or feeling
6. Irregular heart beat
7. Sweating
8. Panic attacks
9. Cough
10. Fatigue
11. other_________________

6. Do you know someone in your area who is using synthetic cannabinoids?

1. Yes, a few people.
2. Yes, up to 10 people.
3. More than 10 people
4. No one

7. Do you think synthetic cannabinoids are more dangerous than marijuana?

1. Yes
2. No

8. Do you think that synthetic cannabinoids use is widespread in our region?

1. Yes
2. No

9. Do you think that synthetic cannabinoids can be used for

Treatment?

1. Yes
2. No

10. What synthetic cannabinoids can you list:

___________________________________

Thank you for your time.
